# Tolerability of Capsaicinoids from* Capsicum* Extract in a Beadlet Form: A Pilot Study

**DOI:** 10.1155/2016/6584649

**Published:** 2016-03-15

**Authors:** Jayant Deshpande, Shankaranarayanan Jeyakodi, Vijaya Juturu

**Affiliations:** ^1^OmniActive Health Technologies Ltd., PEI, Canada C1A 8R8; ^2^OmniActive Health Technologies Ltd., Thane 400 604, India; ^3^OmniActive Health Technologies Inc., Morristown, NJ 07960, USA

## Abstract

A single center, open-label, dose-finding adaptive study was conducted in twelve healthy overweight female subjects. The study was to evaluate the safety and tolerability of the capsaicinoids (CAPs) from* Capsicum* extract in a beadlet form compared to placebo in a healthy overweight population. The investigational product capsaicinoids (CAPs) from* Capsicum* extract in a beadlet form (Capsimax®) a proprietary encapsulated form of *Capsicum* extract in beadlet form supplemented at 2 mg, 4 mg, 6 mg, 8 mg and 10 mg of CAPs. An ascending dose protocol evaluated a total dose of 10 mg daily given in five divided doses (2 mg, 4 mg, 6 mg, 8 mg and 10 mg of CAPs). Each dose was given for a week. Safety and tolerability were assessed. Primary outcomes were tolerability assessments and reports of adverse events. Tolerability assessments were observed on skin color and any changes in skin, bowel movement, digestion, mouth or throat, hair color or changes in hair color, urination includes frequency and burning sensations, breathing, any changes in their health. Secondary outcomes were body weight, body mass index (BMI), blood pressure (SBP/DBP), vital signs, electrocardiograms, clinical chemistry parameters including liver function tests, lung function tests and kidney function tests and complete blood count (CBC). No dose effective changes were observed. The escalating dose levels of CAPs in a beadlet form product found was tolerable and safe for weight management studies. Tolerability assessments and safety blood markers showed no significant changes from baseline. No significant serious adverse events were reported throughout the duration of the study. Further longer term studies are required to explore the tolerability of the product. This trial is registered with ISRCTN: #  ISRCTN10975080.

## 1. Introduction

Obesity rate increased alarmingly in U.S. by 27.7 percent in 2014 [1]. Increased morbidity from diabetes, hypertension, cardiovascular disease, and inflammatory disorders is common in developing and developed countries [2, 3]. More than one-quarter of health care costs related to obesity and associated disorders. Promoting weight loss, decreasing body fat, maintenance of negative energy balance, reducing energy intake, and increasing energy expenditure through physical activity reduce body weight [1]. The interest in natural herb supplements such as* Gymnema sylvestre*, green tea,* Sphaeranthus indicus*,* Garcinia mangostana*,* Dolichos biflorus*, and* Piper betle leaves* for treatment of obesity was rapidly growing because of their minimal side effects. The spicy varieties of* Capsicum* were investigated for their effects on weight loss and thermogenesis. Commonly called chili peppers, or simply “chilies,”* Capsicum* is used as a food additive in many regions due to its pungency, aroma, and color [4]. It was safely consumed in large amounts in many countries, especially in Mexico and Korea, where* per capita* consumption reaches 15 grams per day. More moderate consumption up to 5 grams daily* per capita* of* Capsicum* was reported in Thailand and India. In U.S., consumption of all peppers increased, from an average of 6.94 kg per person in 2005 to 8.66 kg per person in 2012 and consumption of bell peppers grew from 4.17 kg to 5.03 kg, while chili pepper consumption grew from 2.77 kg to 3.36 kg [5]. The daily consumption of all peppers was approximately 24 g/person and chili pepper was 9 g/person.* Capsicum* (*Capsicum annuum* L. or* Capsicum frutescens* L.) and paprika (*Capsicum annuum* L.) were generally recognized as safe (GRAS) for their intended use in food (21 CFR 182.10; 582.10; 21 CFR 182.20; 582.20; 21 CFR 73.340) [6].

The most abundant forms of capsaicinoids found in hot red peppers are capsaicin (trans-8-methyl-N-vanillyl-6-nonenamide), dihydrocapsaicin, and nordihydrocapsaicin. About 70% of the burning sensation experienced from consumption of chili red peppers is attributed to capsaicin [7]. Capsaicinoids (CAPs) are detected rapidly using gas chromatography or supercritical fluid extraction and supercritical fluid chromatography (SFE/SFC). Approximately 3 mg of capsaicinoids is present within 1 g of dried red pepper. Capsaicin is used extensively as treatment for pain. Evidences showed that capsaicin has effects on digestive tract, regulating homeostasis and other deleterious effects. The TRPV1 channels are expressed in many neurons in the GI tract [6].* Capsicum* and capsaicinoids improve metabolism and hormone function [8], stabilize blood glucose [9, 10], reduce insulin and leptin resistance [11] and endothelial function [12], inhibiting LDL-cholesterol oxidation [13], and prevent cancer due to antioxidant activity [14–16]. Spicy food consumption showed highly consistent inverse associations with total mortality among both men and women after adjustment with potential risk factors. The adjusted hazard ratios for death were 0.90 (95% confidence interval 0.84 to 0.96), 0.86 (0.80 to 0.92), and 0.86 (0.82 to 0.90) for those who ate spicy food 1 or 2, 3 to 5, and 6 or 7 days a week, respectively. People consuming spicy foods 6 or 7 days a week showed a 14% relative risk reduction in total mortality. Inverse associations were also observed for deaths due to cancer, ischemic heart diseases, and respiratory diseases [15].

Capsaicinoids (CAPs), potential ingredient to support body weight, reduce* ad libitum* energy intake [17–19], increase thermogenesis and energy expenditure [7, 9, 20–22], and increase lipolysis [7, 9, 20, 21, 23–26]. Since studies have used a variety of treatments (chilies and dietary supplements) within the research design, the results for a given outcome measure the ingested treatment, dose, and product quality. However, assuming the overall effects as noted above, epidemiological data seem to support an association between consumption of CAPs containing foods and a lower incidence of obesity and associated disorders [15, 27].

**Figure 1:**
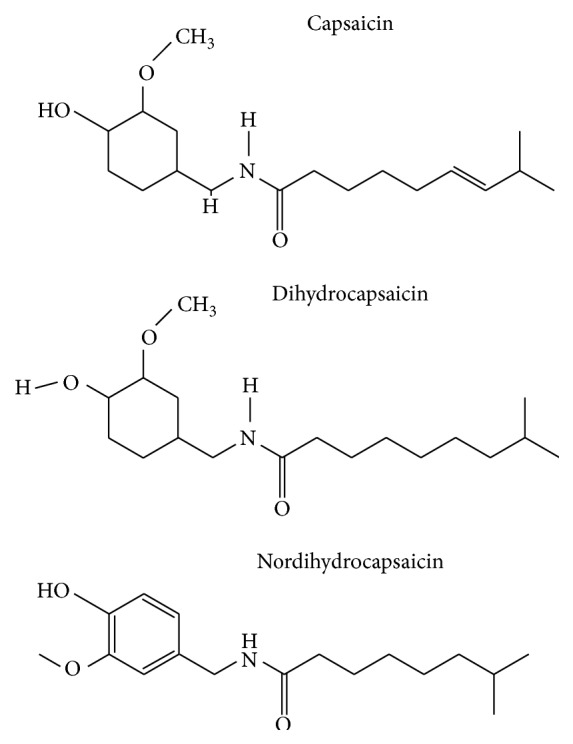
The structures of capsaicinoids.

CAPs are major naturally occurring pungent principles in red-hot pepper (Figure 1) [28]. The current study was a pilot study designed to study the safety and tolerability of capsaicinoids from* Capsicum* extract fruit in a beadlet form, administered to a group of 12 healthy overweight female subjects to escalating dosages of CAPs from* Capsicum* extract fruit (i.e., 2–10 mg capsaicinoids). Each dose is administered for a week (7 days). Daily diary data was maintained by subjects to show general tolerability and record adverse events.

## 2. Materials and Methods

### 2.1. Subjects

The study is designed to test different doses of CAPs product (2 mg CAPs, 4 mg CAPs, 6 mg CAPs, 8 mg CAPs, and 10 mg CAPs) on tolerability, safety, and anthropometric and metabolic measures in overweight healthy subjects. The study was a single center, dose-finding, open-label, and adaptive study design with twelve (12) overweight women taking escalating doses of the study product, each dose for six (6) weeks. An ascending dose protocol evaluated a total dose of up to 10 mg daily given in different doses (2 mg, 4 mg, 6 mg, 8 mg, and 10 mg of CAPs). Each dose is administered to subjects for a week (7 days). Safety and tolerability are assessed by recording vital signs, electrocardiograms, clinical chemistry parameters, urinalysis, and adverse events. The study was conducted at Medicus-Staywell Clinical Research, Northridge, CA. Institutional review board (IRB) approval was received (Copernicus Group, IRB, Cary, NC). All experimental procedures were performed under the Helsinki Declaration. All participants completed a detailed medical history, prior and concomitant medications, and physical activity questionnaire.

*Inclusion and Exclusion Criteria*. Healthy female subjects between 25 and 55 years of age and with body mass index (BMI) between 25 and 34.9 kg/m^2^ were included. All participants signed informed consent forms. Subjects agreed to all study visits and procedures.

Pregnant and lactating women were not included. Subjects were excluded based on regular ingestion of chili peppers (raw or powdered form), black pepper, or ginger or foods known to contain chili peppers, black pepper, or ginger more than 3 times per week. Subjects with known allergies to foods were excluded. Subjects were excluded for any prescribed medications for chronic diseases such as inflammatory disorders, metabolic disorders, diabetes, cardiovascular disease, cancer, HIV/AIDS, and obesity. Subjects with recent history of alcoholism (within 12 months) or drug abuse were excluded.

**Figure 2:**
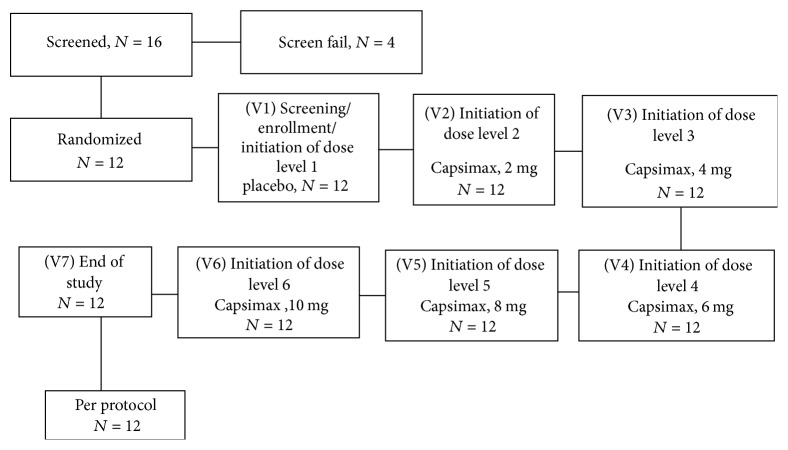
Study design.

Study design and number of visits of participants are reported in Figure 2. Medical history, review of concomitant medications, vital signs, demographic assessments, anthropometric measurements, physical examination, and laboratory assessments including urine collection for pregnancy and blood draws for CBC and CMP were assessed. Subjects were given study product and instructions (Visit 1).

Subjects returned to the site every week and tolerability reviews were conducted using open-ended questions. Subjects fasting samples were collected at baseline and after 6 weeks of product administration. Subject's diaries were reviewed to record AEs. Subjects were given 2 mg CAPs (100 mg Capsimax), 4 mg CAPs (200 mg Capsimax), 6 mg CAPs (300 mg Capsimax), 8 mg CAPs (400 mg Capsimax), and 10 mg CAPs (500 mg Capsimax) during each visit. Each dose was administered for a week (7 days). Vital signs, medical history, and tolerability survey were recorded for subjects at each visit. Blood samples were analyzed for safety markers such as complete blood count (CBC) and comprehensive metabolic panel (white blood cells, red blood cells, hemoglobin (Hb), hematocrit, mean platelet volume (MPV), mean corpuscular volume (MCV), red cell distribution width (RDW), platelet count, mean corpuscular hemoglobin, neutrophil, lymphocyte, monocytes, eosinophil, basophil, total protein, albumin, globulin, ratio of albumin and globulin, calcium, chloride, sodium potassium, and anion gap) and liver, lung, and kidney function tests (serum glutamic-oxaloacetic transaminase (SGOT), serum glutamic-pyruvic transaminase (SGPT), alkaline phosphatase, bilirubin, carbon dioxide, blood urea nitrogen (BUN), and creatinine blood urea nitrogen). Frequency and intensity of adverse event (AE) and serious AEs were recorded based on the diaries and interviews during each visit.

### 2.2. Product Information

Capsimax, a proprietary product, consists of capsaicinoids (CAPs) obtained from dried red fruits of* Capsicum annuum* L. The* Capsicum* extract was standardized into a beadlet form with food grade carbohydrates useful for food applications. Capsimax is a faint, pinkish-white colored, free-flowing, uniform spheroidal beadlet with a spicy odor characteristic of dried ripe fruits of* Capsicum*. The novel delivery technology masks the irritating effects of CAPs in GI. The product was standardized to 2% capsaicinoids, of which 1.2–1.35% is capsaicin, 0.6–0.8% is dihydrocapsaicin, and 0.1–0.2% is nordihydrocapsaicin. The product has 15–25% extract from* Capsicum*, 45–55% sucrose, and 30–35% cellulose gum coatings. Placebo and Capsimax capsules (providing 2 mg, 4 mg, 6 mg, 8 mg, and 10 mg CAPs) from* Capsicum* extract in beadlet form are provided by OmniActive Health Technologies Ltd., India. The placebo capsules were same in appearance and consisted of cellulose. The capsules were manufactured under GMP conditions (Mumbai, India).

### 2.3. Statistical Analysis

A modified per-protocol analysis was performed including all subjects who had at least one poststudy product exposure visit. Mean baseline values and change from baseline were determined. Data are expressed as mean  ±  standard deviation unless otherwise specified. Primary endpoints were tolerability assessments and AEs and secondary end points were blood safety markers (complete blood count (CBC) and comprehensive metabolic panel (CMP)). Change in assessments for tolerability and safety measures is assessed by ANOVA in a repeated measures design. This allowed for an assessment of the main effect of time, dose, and time by dose interaction. A paired sample *t* test was used to assess changes over time for each group. Mean, standard deviation and significance and categorical variables are summarized as counts and percentages. Data derived from diary entries, clinical chemistry assessments, questionnaires, and other relevant assessments at baseline and after supplementation. Statistical Software for Social Sciences (SPSS version 19) was used to run all analysis. Results are considered statistically significant at *p* < 0.05.

## 3. Results

### 3.1. Descriptive Characteristics

Table 1 provides descriptive baseline characteristics of the participants. In the current study, 58% subjects were Hispanics and 42% were non-Hispanic subjects. Overweight healthy women participated in the study (mean BMI: 28 kg/m^2^). Blood pressure was normal at baseline and at postsupplementation.

### 3.2. Tolerability Study

Twelve female subjects participated in the study. All participants completed the study. The primary endpoints were daily diary tolerability assessments and reports of adverse events. Subjects tolerability survey reports suggest that escalating doses of CAPs (2–10 mg CAPs) had no significant changes in the skin, bowel movements, hair, digestion, urination, mouth or throat, and breathing. At week 2, only 92% of the subjects reported no changes in their urination. Subjects reported no changes in their overall health. Four incidences of adverse events such as sprained ankle, elevated blood pressure, stomach ache, and increased urination are reported. However, in investigator evaluation, these incidences unrelated to product consumption. All subjects completed the study and no dropouts were reported. No serious adverse events were reported.

### 3.3. Anthropometric Measurements and Vital Signs

No significant changes were observed for vital signs, body weight, body mass index, and blood pressure over baseline (SBP/DBP, Table 2). CAPs dose escalation did not affect blood pressure.

### 3.4. Blood Chemistries (CBC and Comprehensive Metabolic Panel)

No statistically significant changes were observed in CBC compared to baseline for all markers (Table 3). There were no significant changes in liver, lung, and kidney function test. No significant changes were observed in comprehensive metabolic panel (Table 4). CBC and CMP are within normal range. Repeated measures analysis of variance for all assessments revealed no significant effect of dose on tolerability assessments and safety measures. Overall compliance was ≥91%–100%.

## 4. Discussion

**Figure 3:**
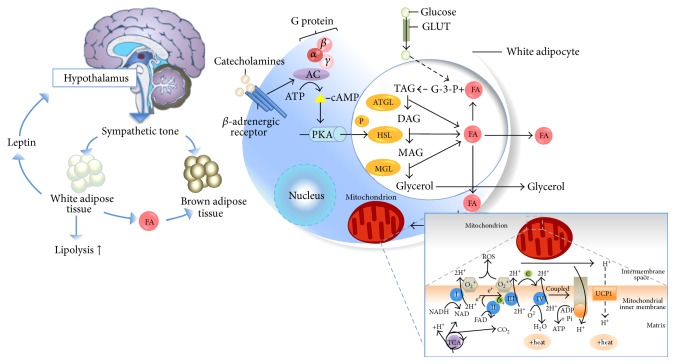
Potential mechanism of action; capsaicinoids promote lipolysis of fat and stimulate thermogenesis in adipose tissue.

Capsaicinoids (CAPs) serve as agonists of the transient receptor potential vanilloid subfamily member 1 (TRPV1). TRPV1 releases substance P, which activates the postsynaptic receptor of substance P, neurokinin-1 [29]. CAPs are found to have weight loss properties based on their potential mechanism of action (Figure 3). Activation of neurokinin-1 results in an increased activation of the sympathetic nervous system, leading to the release of epinephrine (EPI) and norepinephrine/noradrenaline (NA). Recent systematic review and met analysis [30, 31] reported that consumption of 2 mg capsaicinoids increases energy expenditure (50 kcal/day) and would produce clinically significant levels of weight loss in 1-2 years. It was also observed that regular consumption reduced much abdominal adipose tissue levels and reduced appetite and energy intake as part of a weight management program [32].

*In vitro* dissolution data (unpublished) for 500 mg Capsimax beadlets is equal to 10 mg CAPs tested in acidic medium similar to stomach pH. No release of CAPs is observed under acidic conditions. In addition, CAPs from Capsimax were released in alkaline medium, which is similar to conditions in the upper intestine. The release of CAPs in alkaline medium was gradual, over the period of 4 hours. At 4 hours, 75% of the CAPs were released [33]. Results from this study showed that 6-week consumption of the study product in increasing doses (2–10 mg/d) has no significant effect on change in skin color, bowel movements, hair, digestion, mouth or throat, and breathing including urination. There were no significant changes in anthropometric measures, clinical chemistries, CBC, metabolic measures, and vital signs compared to baseline measurements. Lastly, there were no serious adverse events related to the study product. Overall, the results of the study indicated that CAPs supplemented dosages were safe and well tolerated.

The current data supports that higher dose of CAPs was safe and tolerable for human consumption. The results reinforce animal and human studies on the acute effects of CAPs on metabolic measures such as white blood cells (WBCs), neutrophils, eosinophils, basophils, monocytes, lymphocytes, T lymphocytes, B lymphocytes, and NK cells. Capsaicinoids decreased the levels of acquired immunity cells and increased the number of total WBCs and neutrophils without changing the number of monocytes, eosinophils, or basophils [34]. This indicates that intake of CAPs does not elicit harmful immune response.

*In vitro* and* in vivo* studies further support its safety and tolerability [35–38]. No gross or microscopic lesions were observed in any of the rats. The rats showed oral irritation when fed with normal* Capsicum* extract during pilot experiments, because no irritation was noted with CAPs (Capsimax beadlets). CAPs and CAPs plus formulations did not show any adverse effects on body weights or on organs as evaluated by necropsy and histopathological examination. The CAPs (Capsimax) and CAPs containing formulations were tolerable in experimental animals and without any behavior changes. In mutagenesis test, no real increase in revertant colony numbers in any of the five tester strains was observed following CAPs treatment at any concentration, regardless of metabolic activation (S9 mix). There was also no tendency for higher mutation rates with increasing concentrations in the range below the border of biological relevance. The results of these investigations revealed that CAPs did not induce gene mutations by base pair changes or frameshifts in the genome of the strains. CAPs supplementation does not cause mutagenesis. The results of these investigations suggest that CAPs were nonmutagenic in both the presence and the absence of metabolic activation. In the chromosomal aberration test, CAPs were tested in human peripheral blood lymphocyte cultures. CAPs did not induce gene mutations by base pair changes or frameshifts in the genome of the strains used [37]. The results suggest that CAPs supplementation does not cause mutagenesis. CAPs were well tolerated, with no observed side effects related to gastric upset or discomfort. No difference in heart rate and systolic or diastolic blood pressure was noted as compared to placebo at up to 4 hours after dosing [39]. The current findings suggest that daily consumption of capsaicinoids up to 10 mg was well tolerated and no significant changes in blood chemistries were observed. The limitations of the study were only up to 10 mg capsaicinoids for 7-day intervention for tolerability and safety of the product. This study is only trying to find the dose of tolerability to use in efficacy studies.

Capsaicinoids (CAPs) from* Capsicum* extract blended with other ingredients. Recent studies suggest that capsaicinoids from* Capsicum* extract alone and/or with other ingredients reported no adverse events and further confirmed that CAPs are safe [26, 39–45]. The results of these clinical trials show that 2 mg CAPs (Capsimax) were safe and tolerable for human consumption. Furthermore, no serious adverse effects were reported, and the regimen was well tolerated. The current findings suggest that daily consumption of 2 mg CAPs and up to 10 mg CAPs were well tolerated and no significant changes in liver and kidney functions. Further long-term safety and efficacy studies are warranted.

## 5. Conclusions

The escalating dose levels of CAPs from Capsimax product, a highly concentrated natural* Capsicum* fruit extract, were found tolerable and safe for human consumption.

## Figures and Tables

**Table 1:** Baseline characteristics of the subjects.

Baseline characteristics	Subjects details
*N*	12 females
Age (Mean ± SD)	43.0 ± 8.18
BMI (Mean ± SD)	28.7 ± 2.95
Marital status	
Single, *N*	6
Married, *N*	5
Divorced, *N*	1
Ethnicity	
Hispanic, *N*	7
Non-Hispanic, *N*	5

**Table 2:** Body mass index and blood pressure.

Details	Baseline	Placebo	Capsaicinoids from Capsimax				
			Mean ± SD				
	Mean ± SD	Mean ± SD	2 mg	4 mg	6 mg	8 mg	10 mg
BMI	28.68 ± 3	28.65 ± 3	28.76 ± 3	28.69 ± 3	28.77 ± 3.15	28.67 ± 3	28.71 ± 3
SBP	122 ± 17	115 ± 15	118 ± 20	115 ± 15	119 ± 16	117 ± 15	122 ± 21
DBP	74.83 ± 10	71.17 ± 8	72.33 ± 8	69.67 ± 6	75.75 ± 9	74 ± 10	78 ± 7

No significant changes were observed.

**Table 3:** Blood chemistries at baseline and week-6 visits.

Blood chemistries	Baseline	Week 6
	Mean ± SD	Mean ± SD
White blood cells (WBCs)	6.81 ± 1.393	6.68 ± 1.872
Red blood cells (RBCs)	4.44 ± 0.245	4.43 ± 0.327
Hemoglobin (Hb)	13.28 ± 0.783	13.15 ± 1.028
Hematocrit	39.43 ± 2.249	39.08 ± 3.082
Mean corpuscular volume (MCV)	88.92 ± 3.622	88.33 ± 3.580
Mean corpuscular hemoglobin	29.93 ± 1.207	29.74 ± 1.218
Red cell distribution width (RDW)	14.13 ± 1.508	13.87 ± 1.034
Platelet count	235.25 ± 38.973	237.33 ± 48.095
Mean platelet volume (MPV)	9.53 ± 0.859	9.70 ± 0.966
Neutrophil	60.83 ± 11.352	62.00 ± 10.600
Lymphocyte	30.00 ± 10.036	29.50 ± 9.100
Monocytes	6.50 ± 1.679	5.83 ± 1.586
Eosinophil	2.67 ± 1.923	2.25 ± 1.545
Basophil	0.25 ± 0.452	0.42 ± 0.515
Total protein	7.08 ± 0.425	6.98 ± 0.282
Albumin	4.25 ± 0.323	4.20 ± 0.280
Globulin	2.83 ± 0.439	2.78 ± 0.359
Ratio of albumin and globulin	1.55 ± 0.332	1.55 ± 0.265
Calcium	9.33 ± 0.440	8.89 ± 0.578
Chloride	105.58 ± 2.193	104.50 ± 2.844
Sodium	140.00 ± 2.558	138.75 ± 1.960
Potassium	3.99 ± 0.334	3.86 ± 0.318
Anion gap	8.42 ± 1.975	7.58 ± 2.353

**Table 4:** Serum markers for liver, lung, and kidney functions.

Markers	Baseline	Week 6
	Mean ± SD	Mean ± SD
Serum glutamic-oxaloacetic transaminase (SGOT)	18.58 ± 5.178	20.50 ± 7.657
Serum glutamic-pyruvic transaminase (SGPT)	24.33 ± 12.630	26.00 ± 14.610
Alkaline phosphatase	72.17 ± 18.556	76.92 ± 26.387
Bilirubin	0.46 ± 0.202	0.53 ± 0.336
Carbon dioxide	26.00 ± 2.296	26.67 ± 3.085
Blood urea nitrogen (BUN)	11.67 ± 3.143	11.08 ± 3.343
Creatinine	0.72 ± 0.083	0.77 ± 0.078
Blood urea nitrogen	16.33 ± 4.163	14.42 ± 3.554
